# Supplementary material for: Cellular and Humoral Immunity after the Third Vaccination against SARS-CoV-2 in Hematopoietic Stem-Cell Transplant Recipients
Source: Vaccines (Basel). 2022 Jun 18;10(6):972. doi: 10.3390/vaccines10060972 (PMC9230894; doi:10.3390/vaccines10060972)
Supplement: Supplementary file 1 [file vaccines-10-00972-s001.zip › vaccines-1761231-supplementary.pdf]

Table S1. Spearman analysis of cellular immune response in hematopoietic stem cell transplant (HSCT) patients before and after the third vaccination. In each case, spots increment upon stimulation with SARS-CoV-2 proteins/peptide mixtures was correlated with the age of the volunteers.

Antigens tested in *in house* ELISpot vs. age HSCT patients	Correlation coefficient r before third vaccination	*p*-value before third vaccination	Correlation coefficient *r* after third vaccination	*p*-value after third vaccination
S1/S2 IFN-γ	-0.4	0.08	-0.4	0.1
S1 IFN-γ	-0.2	0.4	-0.4	0.09
S1 Sino IFN-γ	-0.1	0.5	-0.5	0.03
S1/S2 IL-2	-0.2	0.4	-0.3	0.2
S1 IL-2	-0.2	0.5	-0.3	0.2
S1 Sino IL-2	0.03	0.9	-0.4	0.1
S-Pool IFN-γ	-0.2	0.6	-0.4	0.2
S-Pool IL-2	-0.3	0.3	-0.7	0.008
S-Pool IFN-γ and IL-2	-0.2	0.5	-0.6	0.03

Table S2. Spearman analysis of cellular immune response in healthy controls before and after the third vaccination. In each case, spots increment upon stimulation with SARS-CoV-2 proteins/peptide mixtures correlated with the age of the volunteers.

Antigens tested in *in house* ELISpot vs. age healthy controls	Correlation coefficient r before third vaccination	*p*-value before third vaccination	Correlation coefficient *r* after third vaccination	*p*-value after third vaccination
S1/S2 IFN-γ	-0.4	0.1	-0.8	<0.0001
S1 IFN-γ	-0.3	0.2	-0.6	0.01
S1 Sino IFN-γ	0.2	0.4	-0.4	0.05
S1/S2 IL-2	-0.5	0.1	-0.3	0.2
S1 IL-2	-0.4	0.2	-0.3	0.2
S1 Sino IL-2	-0.8	0.1	-0.1	0.7
S-Pool IFN-γ	0.2	0.6	-0.2	0.4
S-Pool IL-2	-0.5	0.2	-0.4	0.1
S-Pool IFN-γ and IL-2	-0.01	0.9	-0.4	0.09

Table S3. Spearman analysis of humoral immune response in hematopoietic stem cell transplant (HSCT) patients before and after the third vaccination. In each case, antibody ratio, antibody concentration and titer of neutralizing antibodies were correlated with the age of the volunteers.

Antobides tested vs. age HSCT patients	Correlation coefficient r before third vaccination	*p*-value before third vaccination	Correlation coefficient *r* after third vaccination	*p*-value after third vaccination
Antibody ratioγ	-0.05	0.8	-0.08	0.8
Antibody concentration [BAU/ml]	0.01	0.9	-0.02	0.9
1/Neutralizing Titer	0.02	0.9	-0.5	0.06

Table S4. Spearman analysis of humoral immune response in healthy controls before and after the third vaccination. In each case, antibody ratio, antibody concentration and titer of neutralizing antibodies were correlated with the age of the volunteers.

Antobides tested vs. age healthy controls	Correlation coefficient r before third vaccination	*p*-value before third vaccination	Correlation coefficient *r* after third vaccination	*p*-value after third vaccination
Antibody ratioγ	0.002	0.9	-0.3	0.2
Antibody concentration [BAU/ml]	0.1	0.7	-0.4	0.08
1/Neutralizing Titer	-0.08	0.8	-0.4	0.05

Table S5. Spearman analysis of spots increment in hematopoietic stem cell transplant (HSCT) patients before and after the third vaccination. In each case, spots increment upon stimulation with SARS-CoV-2 proteins/peptide mixtures was correlated with the interval between HSCT and blood collection.

Interval between HSCT and blood collectionvs. spots increment in HSCT patients	Correlation coefficient r before third vaccination	*p*-value before third vaccination	Correlation coefficient *r* after third vaccination	*p*-value after third vaccination
S1/S2 IFN-γ	0.2	0.4	-0.2	0.4
S1 IFN-γ	0.1	0.5	-0.3	0.2
S1 Sino IFN-γ	-0.1	0.6	-0.5	0.03
S1/S2 IL-2	-0.04	0.9	-0.2	0.5
S1 IL-2	-0.1	0.6	-0.03	0.9
S1 Sino IL-2	-0.07	0.8	-0.2	0.4
S-Pool IFN-γ	-0.08	0.8	-0.2	0.5
S-Pool IL-2	0.07	0.9	-0.1	0.6
S-Pool IFN-γ and IL-2	-0.1	0.7	-0.2	0.5

Table S6. Spearman analysis of antibodies tested in hematopoietic stem cell transplant (HSCT) patients before and after the third vaccination. In each case, antibody ratio, antibody concentration and titer of neutralizing antibodies were correlated with the interval between HSCT and blood collection.

Interval between HSCT and blood collectionvs. Antibodies tested in HSCT patients	Correlation coefficient r before third vaccination	*p*-value before third vaccination	Correlation coefficient *r* after third vaccination	*p*-value after third vaccination
Antibody ratioγ	0.02	0.9	-0.1	0.6
Antibody concentration [BAU/ml]	0.04	0.9	-0.4	0.1
1/Neutralizing Titer	0.1	0.6	-0.1	0.6

Table S7. *P*-values of two-tailed Mann-Whitney tests comparing the results of HSCT patients and healthy controls before and after third vaccination.

Antigens tested in CoV-iSpot	HSCT pts. vs. controls before third vaccination	HSCT pts. vs. controls after third vaccination
S-Pool (IFN-γ)	0.8	0.4
S-Pool (IL-2)	0.1	0.007
S-Pool (IFN-γ + IL-2)	0.6	0.02

Table S8. *P*-values of two-tailed Mann-Whitney tests comparing the results for IFN-γ of HSCT patients and healthy controls before and after third vaccination.

Antigens tested in *in house* IFN-γ ELISpot	HSCT pts. vs. controls before third vaccination	HSCT pts. vs. controls after third vaccination
S1/S2	0.07	0.08
S1	0.01	0.006
S1 Sino	0.1	0.001

Table S9. *P*-values of two-tailed Mann-Whitney tests comparing the results for IL-2 of HSCT patients and healthy controls before and after third vaccination.

Antigens tested in *in house* IL-2 ELISpot	HSCT pts. vs. controls before third vaccination	HSCT pts. vs. controls after third vaccination
S1/S2	0.1	0.0005
S1	0.04	0.01
S1 Sino	0.02	0.02

Table S10. *P*-values of two-tailed Mann-Whitney tests comparing the results of HSCT patients and healthy controls before and after third vaccination.

Mutations/variants tested in neutralization antibody assay	HSCT pts. vs. controls before third vaccination	HSCT pts. vs. controls after third vaccination
Wild type S1	0.4	0.002
Wild type RBD	0.5	0.002
Alpha S1	0.6	0.0008
Beta S1	0.5	0.0008
Gamma RBD	0.7	0.0008
E484K RBD	0.4	0.0008
Epsilon RBD	0.5	0.0008
Kappa RBD	0.5	0.0008
D614G S1	0.5	0.002
K417N RBD	0.5	0.002
N501Y RBD	0.4	0.0008

Table S11. *P*-values of two-tailed Mann-Whitney tests comparing the results of HSCT patients and healthy controls before and after third vaccination.

Antibody ELISA	HSCT pts. vs. controls before third vaccination	HSCT pts. vs. controls after third vaccination
Antibody Ratio	0.6	0.002
BAU/ml	0.5	0.2
